# A qualitative study of experiences of health and social care in home mechanical ventilation

**DOI:** 10.1002/nop2.213

**Published:** 2018-11-10

**Authors:** Jessica MacLaren, Pam Smith, Sheila Rodgers, Anthony P. Bateman, Pam Ramsay

**Affiliations:** ^1^ University of Edinburgh Old Medical School Teviot Place Edinburgh UK; ^2^ Present address: School of Health and Social Care Edinburgh Napier University Edinburgh UK

**Keywords:** care experiences, Duchenne muscular dystrophy, family carers, health and social care integration, home mechanical ventilation, motor neurone disease, qualitative research, semi‐structured interviews

## Abstract

**Aim:**

To contribute insight into health and social care integration through an exploration of the care experiences of adults with degenerative neuromuscular conditions who use a mechanical ventilator at home.

**Design:**

Descriptive qualitative research.

**Methods:**

Seventeen semi‐structured interviews were conducted with patients and family carers living in Scotland during 2015–2016 and thematically analysed.

**Results:**

To achieve a satisfying life, home ventilated participants required help from a variety of health and social care services, as well as care from family. Examples of successful care were identified, but there were also serious failures and conflict with services. Identifying how care fails or succeeds for this patient population and their families requires an understanding of the interdependency of health and social care. This was achieved by examining health and social care provision from the experiential perspective of care‐users to provide insights into how disconnected provision has an impact on users’ lives in numerous, idiosyncratic ways.

## 1 INTRODUCTION

The interdependency of health and social care is well established (Avendano & Kawachi, 2014; Bradley, Elkins, Herrin, & Elbel, 2011; Winters, Magalhaes, Kinsella, & Kothari, 2016) and there is a major political drive towards integration of health and social care services across the European Union (European Social Network, 2017; Scottish Government, 2015). However, this way of working challenges traditional models of health care and often fails in practice (Glasby & Dickinson, 2014). Furthermore, discussions of health and social care integration tend to focus on systemic or structural issues and the perspective of individual services users can get lost (Nuño, Coleman, Bengoa, & Sauto, 2012; Winters et al., 2016). Kodner and Spreeuwenberg (2002) argue that the understanding of “integration” should instead centre on the patient’s perspective and experience of care. In this paper, findings from research with adults who have Duchenne/Becker muscular dystrophy (D/BMD) or motor neurone disease (MND) and who use home mechanical ventilation (HMV) provide a personal perspective on these issues, describing the everyday experiences of receiving care from multiple services.

The aetiology of D/BMD and MND differs, but patients face a common set of challenges in terms of living with similar types of disability. Both are rare conditions, MND affects 4.02–4.91 per 100,000 people (Hoppitt et al., 2011) and D/BMD 1 in every 3,500 male births (Romitti, 2015). Population numbers are therefore small, but the total societal costs associated with these conditions are high (Landfeldt et al., 2014). Respiratory complications are the most common cause of death for people MND or D/BMD and the development of HMV has contributed to a longer life expectancy and better quality of life for patients (Annane, Orlikowski, Chevret, Chevrolet, & Raphaël, 2014; Eagle et al., 2002; Simonds, 2006). HMV provision across nations is inequitable (Dybwik, Tollåli, Nielsen, & Brinchmann, 2010; Mandal et al., 2013), but from their 2005 study, Lloyd‐Owen et al. estimated that 6.6 per 100,000 people used HMV (including non‐neuromuscular conditions). Relieved of debilitating and life‐limiting chronic hypoxia, there is the potential for people with MND or D/BMD to live more comfortable, active and satisfying lives, but only with adequate care provision.

### 1.1 Background

During the past 20 years, HMV has been implemented in an increasing number of countries, but the care arrangements which support HMV, vary greatly between and within countries (Ando et al., 2015; Hannan et al., 2016; Lloyd‐Owen et al., 2005; Nasiłowski et al., 2015; Stuart & Weinrich, 2001). Individuals using HMV require an organized care structure to facilitate a good quality of life (Stuart & Weinrich, 2004). However, there is little experience focused research into care arrangements for adults with neuromuscular conditions who use HMV. Instead, the majority of studies investigating patient or family perspectives use standardized measures of health related quality of life (HRQoL; cf. Gauthier et al., 2007; Vincent, Carr, Walburn, Scott, & Rose, 2007).

A small number of qualitative (mainly Scandinavian) studies have been conducted into the experiences of adults receiving HMV for various health conditions, (cf. Ballangrud, Bogsti, & Johansson, 2009; Dreyer, Steffensen, & Pedersen, 2010a; Dreyer, Steffensen, & Pedersen, 2010b; Ingadóttir & Jonsdottir, 2006; Laakso, Markström, Havstam, Idvall, & Hartelius, 2014; Lindahl, 2010; Lindahl, Sandman, & Rasmussén, 2005). These confirm the findings of studies using HRQoL (that HMV has a generally positive impact on patients’ lives), but they also uncover new complexities (Ingadóttir & Jonsdottir, 2006). While ventilation enhances wellbeing for many, it requires adaptation and users can encounter practical and social problems (Dreyer et al., 2010a, 2010b). Some find the idea of ventilation prohibitively frightening and poor experiences of services may lead people to decline ventilation (Ando et al., 2015). Becoming dependent on a ventilator can be both life‐saving and also symbolic of death, disease and deterioration (Ballangrud et al., 2009; Lindahl, 2010).

The availability of support and care influences the decision about whether to begin ventilation and ongoing quality of life (Lemoignan & Ells, 2010; Lindahl et al., 2005), but in existing research, descriptions of care for adults with D/BMD or MND who are using HMV are few. Studies either focus on a more diverse population of HMV users or focus on one aspect of care (Ingadóttir & Jonsdottir, 2006; Schaepe & Ewers, 2017). Several studies identify the quality of relationship with carer‐givers as important (Dreyer et al., 2010a, 2010b; Schaepe & Ewers, 2017) and Ballangrud et al. (2009) found feeling in control of one's care was important. Nurses and family members are the most prominent characters in descriptions of care for HMV, but there can also be a need for specialist care requirements like speech therapy (Laakso, Markström, Idvall, Havstam, & Hartelius, 2011). Two Swedish studies describe care as causing suffering for some patients, but also found relationships with carers can enrich the patients’ life (Lindahl, 2010; Lindahl, Sandman, & Rasmussen, 2003, 2006 ). One study focusing on HMV experiences of men with DMD found that patients valued continuity of care and individualized care arrangements (Dreyer et al., 2010a, 2010b).

The provision and accessibility of health and social care for this group of patients varies between and within, countries (Worsley, Telford, & Land, 2014). For example, in some areas, all patients have a personal assistant service (cf. Ballangrud et al., 2009; Dreyer et al., 2010a, 2010b), while in others it is a qualified nurse who provides care (Schaepe & Ewers, 2017). In Scotland, (Ferrie, Wiseman, & Watson, 2013) found that a considerable proportion of care for people with MND is provided by families. The importance of families in caring for adults using HMV is recognized in the literature (Dybwik, Nielsen, & Brinchmann, 2011; Worsley et al., 2014).

To summarize, there is a developing body of research into experiences of HMV, including HMV for neuromuscular diseases like D/BMD and MND. This literature includes some evidence about the kinds of care that these patients receive and how they experience this, although descriptions of care are still few. There is also a need for more evidence about the experiences of family members who care for an adult HMV patient. The nature of care arrangements is a crucial element in life with a ventilator and as Lindahl (2010) and Dybwik, Tollåli, Nielsen, and Brinchmann (2011) argue, care providers need to develop a better understanding of how patients and families experience care. Furthermore, more understanding is needed of how care arrangements in different contexts contribute to the quality of life lived by people using HMV, adding contextualized detail to the picture of care for HMV and D/BMD and MND patients. The study described below asked the following question: How do patients and their family carers experience living with a ventilator at home and how do they experience their care?

## 2 METHODS

### 2.1 Design

Experience‐focused studies on this topic are still few in number and therefore this was an exploratory study with a descriptive qualitative design. The research aimed to develop a picture of daily life lived with a ventilator as experienced by the participants. “Experience” was differentiated from immediate perception and conceptualized as being already interpreted and accounts of experience as communicating meanings that are important to the individual (Ricoeur 1983/1984). The emphasis on daily life responds to the long term, home‐based nature of HMV. HMV for people with D/BMD and MND has the special features of being associated with a high degree of physical impairment, but also functioning as a mundane, everyday part of life. The research set out to understand the unique experience produced through this association between severe illness and normality.

### 2.2 Method

Data were collected between 2015–2016, using semi‐structured interviews conducted by the project researcher who had no prior relationship with the participants. Purposive sampling was used to recruit patients and their families (Table 1 for inclusion and exclusion criteria). Patients were identified and approached by their Respiratory and Home Ventilation physician. The project researcher then contacted those who were interested in participating.

**Table 1:** Inclusion and exclusion criteria

Inclusion criteria (Patients)	Exclusion criteria (Patients)
Individuals over the age of 16 Living in Scotland. Under the care of a specialist HVS. Diagnosed with either MND or B/DMD. Using a ventilator at home with a minimum of overnight use	Individuals who were in crisis. Individuals who lacked capacity

Inclusion criteria (Family members)	Exclusion criteria (Family members)
Family of a patient interviewed for this study. Individuals over the age of 16	Family not undertaking substantial care responsibilities (e.g., not living with the patient). Patient did not want family member to be interviewed. Individuals who were in crisis. Invidiuals who lacked capacity

A total of 20 participants were recruited, including 14 patients, who had a variety of physical capabilities, ventilator usage and care arrangements (Tables 2 and 3). The male‐gendered nature of the patients’ sample reflects the aetiology of these conditions, which mainly or exclusively affect men. A total of six unpaid family carers were also recruited (Table 4).

**Table 2:** Sample of patients

Characteristics of patient participants	N
Male	13
Female	1
Total	14
Age	
Age range (years)	17–73
Mean age (years)	44
Diagnosis	
Motor neurone disease	7
Duchenne muscular dystrophy	6
Becker muscular dystrophy	1
Physical capabilities	
Unable to walk	13
Able to walk with an aid	1
Little or no movement in torso, head, arms or hands	9
Some hand movement	5
Had spinal fusion operation	2
Ventilator usage	
Nightime only use	2
Nightime and occasional daytime use	5
Nightime and regular, frequent daytime use	4
Continuous ventilation	3
Type of ventilation	
Invasive ventilation with tracheostomy	3
Non‐invasive ventilation using face mask	11
Duration of ventilator use	
<1 year of use	3
1–9 years of use	6
10 + years of use	5

**Table 3:** Participants’ care arrangements

Description of basic care arrangements (not including specialist health services like home ventilation or motor neurone disease nurses)	Number of patients
24 hr care package. Care provided by personal assistants, directly employed by the patient and managed by them	2
24 hr care package. Care provided by a small team of carers employed by a care agency	1
In residential care settings with 24 hr care	2
Agency‐employed carers visiting the house several times a day and for several hours a day. Family (mostly partners) also providing several hours’ care a day. One participant also attended a hospice day centre once a week	4
Most care given by parents, with up to a few hours per week from support workers	3
All care given by partner	1
Most care given by partner, with patient attending a day centre every day	1
Total	14

**Table 4:** Sample of family carers

Sample of family carers (Relationship to patient)	N
Parent	1
Partner	5
Total number of carers	6

Participants chose whether they wished to be interviewed with their carer or separately. This produced a dataset containing both solo and dyadic interviews (Table 5). Pragmatically, this had the advantage of facilitating recruitment of a seriously ill population and maximizing the range of data collected (Morgan, Ataie, Carder, & Hoffman, 2013), but the resulting mixture of interview formats also added to complexity of the dataset (already complicated by a non‐homogenous sample). We discuss the implications of this in the analysis section below.

**Table 5:** Interview format

Interview format	
Solo interview with patient	9
Solo interview with carer	3
Dyadic interview with patient and carer	5
Total number of interviews	17

Interviews lasted around 60 min and were loosely structured using a topic guide (Table 6) to invite participants to talk about what was important to them and gain an understanding of the participant’s unique context (Rubin & Rubin, 2005). All interviews took place in the participant’s home by their request. One MND participant was unable to speak and participated in a dyadic interview with their partner, using a voice synthesizing app. Interviews were digitally recorded.

**Table 6:** Interview topic guides

**Patient interview topic guide**	
Topic 1. Everyday life with a ventilator	Starting ventilation Adapting to using a ventilator Daily routine with a ventilator
Topic 2. Care needs and services	Accessing care Organization of care
Topic 3. Living with disability	Equipment and resources needed Social life and activities Feelings about lifestyle and quality of life
**Carer interview topic guide**	
Topic 1. Care	Accessing care Organization of care Daily routine as a family carer
Topic 2. Personal impact of caring	Adapting to being a family carer Effect on relationships (with patient and other family members) Feelings about being a family carer
**Introductory statement and prompts**	
*I'm* * interested in understanding what i* *t's* * like to use a ventilator at home. Can you tell me about that?* *Can you describe for me an average day?* *Can you tell how you came to begin home ventilation?*	

To ensure rigour, the researcher kept field notes to record immediate impressions during data collection and the project team regularly reflected on the analysis. Findings were also discussed at a knowledge exchange event, attended by participants, practitioners and charity representatives. This discussion strengthened credibility, aiding in confirming and refining the findings.

### 2.3 Analysis

Interview recordings were transcribed verbatim. Non‐lexical expressions were removed to create a more readable transcript. Several interviewees used Scots dialect words and these were transcribed using the common spellings (e.g., *cannae = can’t*).

The dataset was complex, but dividing it into sub‐groups (e.g., solo and dyadic interviews, or carers and patients) would have created unbalanced groupings and potentially missed areas of connection and contrast. The dataset was therefore analysed in its entirety looking for emergent meanings in the data (Guest, MacQueen, & Namey, 2012). The interview texts were read multiple times to promote immersion in the data and coded for content. Initially, the analysis looked for themes shared between the interviews and patterns and repetitions and then for anomalies and contrasts between the interviewees (Guest et al., 2012). To enhance reflexivity, analytic reflections were recorded using memo writing and memos were used to feed into development of the themes. NVivo 10 was used to manage the data.

Themes were developed in line with the analytic objective of understanding “how care works, in this context, for this person” (Guest et al., 2012). Analysis was carried out by the researcher and discussed and refined by the project team. In this article, we focus on a major theme emerging from the data that centred on accounts of different aspects of care. Four sub‐themes make up the theme of Care: Complexity; Conflict; Failures and Successes. These are explored below.

### 2.4 Ethics

The study was reviewed by the HRA Research Ethics Committee. Participation was voluntary and confidential and informed consent gained before data collection. All data were anonymized and participants assigned pseudonyms.

The involvement of the patients’ physician in recruiting participants introduced a potential conflict of role and participants were assured that the project researcher had no involvement in their care. Of the 21 patients approached, seven declined to take part. Some volunteered reasons for declining, including a recent worsening of symptoms, current problems with care arrangements and having recently been involved in another research study. This suggests that patients did not feel obligated or coerced into taking part.

The open style of the interviews and the nature of the topic under discussion, meant that highly sensitive subjects could arise, with a risk of emotional distress to the participants. The researcher (a registered mental health nurse), maintained a sensitive approach, providing space for participants to express feelings as they wished. Where appropriate, participants were also offered information about a free counselling helpline.

An emergent ethical challenge for this study was the collection of data from both patients and family carers and issues of conflict and confidentiality between these. Some data collected from carers in solo interviews were highly sensitive and gave details about relationship breakdown with the family member for whom they were caring. These data could cause distress to the patients involved. However, the research team believe that it is important to publish the anonymized data to raise awareness about the problems confronting these family carers, who provide essential support to their relatives, but are arguably undervalued by statutory services.

## 3 RESULTS

When I've got a chest infection or a cold, it's like craving, that I need it, because I cannae get any breath at all. And as soon as I go on it, it's “whoosh!”… It looks like I'm dead… but as soon as I am on [the ventilator] all the blood in my face all comes and I am “whoosh”! I'm alive. (Sam).

All the patients interviewed valued HMV and felt that it made a positive contribution to their lives. As Sam explains above, ventilation could feel miraculous. However, participants’ experiences show that by itself ventilation cannot enable an individual to live a satisfying life and that furthermore, HMV can have far less positive implications for families who are responsible for providing care for the ventilated person. HMV and the home ventilation service (HVS) that provides care for the participants are only one part of a complex machinery of care that is different for each patient and family.

### 3.1 Complexity

All the patients needed help with daily living and required long‐term care, with the majority of day‐to‐day care provided either by unpaid family members or paid social carers. In Table 7, two participants describe the small, but important details (such as the precise positioning of the ventilator mask), that constitute care for them.

**Table 7:** Descriptions of care

**Care from family**
*When i* *t's* * time for bed I have to hoist him… out of his chair into bed, get him all comfy and stuff like that, put his bed up… then make sure the water is fresh in the [humidifier], so I just go empty that out and use boiled water from the kettle, put that on and i* *t's* * just a case of switching the [ventilator] on… I have to put the [nasal pillows] over [Ror* *y's* *] head and it takes a good while for me to get it in the right position because h* *e'l* *l say i* *t's* * too far that way, ther* *e's* * air escaping… but once he gets it right h* *e's* * like tha* *t's* * fine and he will just go off to sleep (Clare)*
**Care from paid social care workers**
*Two hours in the morning, an hour in the afternoon and an hour sort of tuck in evening… They bring the ventilator through during the day if I need it, make sure that i* *t's* * all set up, that the water in the humidifier is topped up… help me feed if I need to… getting dressed, shower, getting up, on and off of the toilet (Fergus)*

While help with daily living was the most significant component of care in terms of face‐to‐face contact time, participants also had varying degrees of contact with other specialist services and together with social care and family care, these formed the care assemblage for each individual. In this section, we map aspects of this complexity by describing three elements of the care assemblage: paid social care, specialist health services and family carers.

### 3.2 Paid social care

The majority of paid, day‐to‐day care was provided by state funded social carers employed through private agencies. Carers have minimal training and provide support with activities of daily living including washing, dressing, eating and mobilizing. Social carers also assisted participants (to varying degrees) with their ventilators, including putting the mask on, switching the ventilator on and off, attaching tubing and assisting with tracheostomy care.

### 3.3 Specialist services

In addition to social care, participants also used specialist health and social services. These included the HVS, palliative care teams and hospices, neurologists and specialist MND nurses. Care was also received from more generic services including district nurses, family physicians and occupational therapists (who were responsible for assessing requirements for equipment in the home). Engagement with such a complex network of services posed considerable challenges for the participants. They felt that services did not always communicate with one another and there was no single service or practitioner who had an overview of their care.

### 3.4 Family carers

Family carers were key in enabling ventilated patients to stay at home by overseeing the implementation of care and ensuring that services were delivered; and health and social services seemed to expect this of families. During one solo carer interview, Fiona described feeling exhausted by the demands of first caring for her partner and then subsequently (once a care package was in place), overseeing the provision of care and acting as a point of contact for services:

This care package does not help me, this care package enables [my partner] to stay at home… It makes my life a lot more difficult… It's quite clear without me in the care package, it would be very difficult to sustain because [the services] have to have me as a last ditch contingency. (Fiona).

This emphasises the crucial role being played by family carers who are the “last ditch contingency” and the emotional, physical and social costs of this role. The family and the patient have the clearest understanding of how care as a whole is working, but this is burdensome for individuals and their families already coping with the consequences of life‐limiting illness.

### 3.5 Conflict

Living with a life‐limiting illness can be “totally debilitating and totally alien and totally horrendous” (Fergus) and as Ben explained, some feelings associated with this can be difficult to express or manage and can lead to conflict in relationships:

I'm putting my life in [my carers’] hands. You may think you're scared. Imagine how I feel, I'm putting my life into your hands… a lot of clients get angry, aggressive, shout. It's fear… (Ben).

Conflict emerges as a feature of relationships with services and family‐patient relationships. This resonates with previous research identifying continual conflict occurring between family carers and services (Dybwik, Tollåli, et al., 2011). In solo carer interviews, both Fiona and Jenny described conflict with their partners, caused by the stresses of coping with their partner's illness:

I've got to the stage now where I don't want to be here and that's not right, I shouldn't feel like this, but that's how I'm feeling… I've not had respite… I've just reached the end of the line and I can do anything [my partner] asks me to do for him but I don't have a conversation. We don't speak much at all. (Jenny).

A picture emerges where families must face not only life‐limiting illness, but the breakdown of a relationship and possibly wanting to leave the relationship. However, both Jenny and Fiona felt that they could not rely on health and social services to provide adequate care if they were not present to oversee this:

It is absolutely shit living with this situation, do you know what I mean? And any compassionate emotional feelings I felt about [my partner's] situation, are probably back 3–4 years ago… I want to make sure he is safe, I want to make sure he is looked after. And I don't want him to end up being turfed off into somewhere like [a care home] or something like that. But to maintain that situation I have to be around… (Fiona).

The most common experience of conflict with services was around accessing funding or equipment. This could feel like “a constant battle” (Fergus) and participants had to be assertive in fighting for the things they needed. Andy (who had been living with a ventilator for twenty‐four years) was active in persuading services to provide equipment he wanted: “they never give you anything easily. Usually you’ve got to fight for everything” (Andy). However, this required determination and expertise, “I worry about people that aren’t quite as confident… or aware of how the system works” (Steve). Both Andy and Steve had been living with DMD for many years and had developed expertise in negotiating care and funding.

In contrast, participants with MND had a steeper learning curve, sometimes journeying from diagnosis to becoming very disabled in a matter of months. Robert described unable to get adaptations to the family home: “[Social services] wouldn't fit me a shower, they wouldn't give me a ramp, “you'll have to move" [they said]” (Robert). The experience of fighting for care or resources was almost universal among the participants. Funding for assistance with daily living was decided on a case‐by‐case basis and varied between different regions. There was a sense that individuals could not depend on receiving funding for an adequate amount of care.

### 3.6 Failures

Interactions with services could be unpleasant and there were reports of poor interpersonal skills on the part of health and social care professionals: “The psychiatrist said to me: ‘Ah you have been sent to me by [the neurologist], because he thinks you might be one of his crazies!’” (Mike). Patients and family encountered misperceptions about the aetiology of their condition, such as believing that people with MND can't feel pain. Some participants faced constant failures in the provision of care:

It's everything, you know, it's any excuse, the whole service as a package. Whether it is ventilation, MND, the chemist bringing your stuff on time. If I could'nae talk, or had my wife to go and phone‐ these tablets are overdue three days. And it's like constant things, no like now and again. (Angus).

For Angus, living with continual failures of care was exhausting making it difficult to trust services. As Ben described, being completely dependent on others means “I need to trust” (Ben). When tasks were poorly performed patients felt insecure “[the carer] came in to bed me down. I nearly fell out of the bed. She wisnae doing it right” (Robert).

Many failures of care involved agency‐employed carers. As in Robert's example, carers were often inadequately prepared to work with this population of patients, including assisting with the ventilator. This in turn was anxiety provoking for the patient, “they're quite nervous of doing anything and I always want [the ventilator] on quite quickly, so I'm getting myself in a fluster” (Angus). Steve recalled that in the past the HVS would provide training for carers in how to use the ventilator, but with increased pressure on the service this was no longer available. It seems significant failures in services exist through poor organization, co‐ordination and lack of knowledge of the needs of the person living with home ventilation.

### 3.7 Successes

Several participants described experiences of care arrangements that were reliable and met their needs. These included: small, stable teams of carers, the MND nurses and the HVS.

### 3.8 Small, stable teams

Having a small, stable team of social carers was an important element of successful care. Two participants received funding to employ their own care teams, recruiting and managing their carers directly, “I advertise for staff myself and I interview them and train them as well” (Andy). Both reported being satisfied with this arrangement, although it required time and effort to manage. Ben and Fergus (both of whom had agency‐employed carers) also identified having a small care team, with low staff turnover, as important:

I have been quite lucky with the company that I got placed with… a wee family run business, so the carers are consistent. You see the same people, you get to know them. (Fergus).

When carers are present in the home for many hours a day, well‐established relationships help to ensure that the atmosphere remains homelike, “I do my own thing, they do their own thing. If I want anything, I shout, because I like my own space… it's my home… I don't want them to treat it like a hospital” (Ben).

### 3.9 The MND nurses

Data concerning specialist MND nurses came from half the sample (there was no equivalent service for men with DMD). Patients valued the information, emotional support and advocacy provided by MND nurses and the nurses had the potential to act as a main point of contact, but caseloads were very large, limiting the time they could spend with patients.

### 3.10 The HVS

Three features of the HVS emerged as important: they visited at home; were easy to contact; and seemed to have a better understanding of patients’ needs than other services. As Mike observes, home visiting is particularly important for people living with a high degree of disability and ensures equity of access to a service:

Once you can't get a clinic without excessive effort, MND neurologists don't visit in the community. So they lose sight of patients. We've had [the home ventilation physician] out seven times in the last three years and not once has my neurologist visited. (Mike).

Ease of contact was also important. Participants could phone or text their own HVS nurse and could contact the team out‐of‐hours. This compared favourably with the time and effort needed to contact other services. However, as numbers of home ventilated patients increased, participants felt that the care provided by the HVS had become more reactive and limited, where originally the service had provided informal extras (such as training for carers) and would proactively check on patients.

Participants felt that other services were less accessible or responsive than the HVS and there was a sense that the HVS really understood their patients’ needs, “I am happier dealing with the Ventilation Team because I feel they know the ins and outs of ventilation and… know my care set up better” (Steve). However, while Fiona said of the HVS “I've always felt they get it”, she pointed out that, “they can't make Social Services provide a joined up effort around the care”. One successful service could not make the care machinery as a whole work well and the lack of oversight over the whole of an individual's care left families and patients to address aspects of care which were not working so well.

## 4 DISCUSSION

Providing care across different services is hugely challenging for organizations and practitioners. It is acknowledged in the integration literature that patients should be at the centre of service provision, but discussions of health and social care integration tend to focus on the systemic/structural perspective, rather than the experiences of those who receive care (Winters et al., 2016). An alternative approach is to explore how care works for specific people in specific contexts. People with D/BMD or MND who use HMV are a relatively recently developed patient population, who are living longer with complex care needs and consequently require long‐term care from a variety of sources. Their experiences offer illuminating insights into how health and social care functions for patients.

Even in a small sample, the picture captured through the research findings above is primarily one of complexity and heterogeneity, where care arrangements are influenced by symptomology, life circumstances and access to resources. *Care* in the participants’ accounts is composed of families, personalities, equipment, houses, money, services, politics and many other things. It is a dynamic machinery that is commodified and divisible for services (Kodner & Spreeuwenberg, 2002; Phillips, 2007), but that is a whole thing in its impact on the care‐user. This creates an irreconcilable tension for HMV patients and their families who are caught between care as a whole‐person‐effect and care as something organized into separate services.

Privileging the experience of patients highlights questions about the differing socio‐economic values assigned to care interventions. For example, the role of family carers remains under‐recognized and under‐supported by services and yet as in previous research (Ferrie et al., 2013), the findings above suggest that families may act as an information conduit and communication point for different services. Are families creating integration of services in the absence of meaningful systemic integration? This is an area that requires further research. Attending to the experience of patients also highlights the essential role of paid social carers in supporting daily life, problematizing how little funding and education are available for these workers. For the participants, there was a clear discrepancy between the excellence of some services/equipment/practitioners and the difficulties experienced with others.

The issues identified around care raise ethical issues for health and social services. HMV technology is now well developed, effective and reliable. HMV has had a significant effect on the lives of people with D/BMD and MND and similar conditions, opening up new possibilities for these people (Annane et al., 2014; Eagle et al., 2002; Simonds, 2006). However, the participants’ experiences show that care is an essential partner to HMV. There is a disjuncture between the provision of ventilators and the provision of the care required to facilitate a satisfying life for someone using HMV. This disconnection is a product of the organization of health and social care and means that patients are offered the possibility of a longer, more satisfying life, without being given the resources they need to achieve this.

There is evidence that care is an important consideration when deciding whether or not to begin ventilation (Lindahl et al., 2005) and HMV services should consider how to address care issues and give a realistic sense of how care will work and what problems may be encountered when discussing HMV with prospective users. The systemic issues identified in this study may seem insoluble for individual practitioners, but practitioners may be able to improve the service they offer patients by simply increasing their understanding of the complexity of care received by these kinds of patients and the importance of the care provided from outside of health and social care systems, such as families and voluntary services.

### 4.1 Limitations

The findings of this study resonate with issues identified in existing research studies conducted with various HMV patient populations and in different systems of health and social care, suggesting that although care systems can vary considerably, these findings can inform understanding of HMV in different countries.

The data collected from carers is drawn from a small sample and only three carers were interviewed alone. This paper highlights striking data from two of the solo carer interviews. These can only be regarded as indicative of issues that require further investigation, but again, there is resonance with issues identified in studies conducted in different care systems.

## 5 CONCLUSION

The idea of health and social care integration presupposes the organization into specialist services that is characteristic of many health and social care systems, but privileging patients’ experiences re‐frames care to be seen as a whole, consisting of interdependent elements. This provides important understandings about what care does and challenges norms about the value assigned to different kinds of care. This study focused on one specific patient population, but the issues raised are relevant to the increasing numbers of people who are living with long‐term conditions and complex care needs, but whose needs are poorly catered for by current care systems.

## PATIENT CONSENT STATEMENT

Informed consent to publish anonymized data from the research was obtained from all patients recruited to the study.

## ETHICAL APPROVAL

The study was approved by the HRA Research Ethics Committee (UK), Ref. 14/SS/1082.

## CONFLICT OF INTEREST

The authors have no conflict of interest to declare.
